# Electrical Behavior of Combinatorial Thin-Film Zr_x_Ta_1−x_O_y_

**DOI:** 10.3390/nano15100732

**Published:** 2025-05-14

**Authors:** Matthew Flynn-Hepford, Reece Emery, Steven J. Randolph, Scott T. Retterer, Gyula Eres, Bobby G. Sumpter, Anton V. Ievlev, Olga S. Ovchinnikova, Philip D. Rack

**Affiliations:** 1 Materials Science & Engineering, University of Tennessee, 1505 Middle Dr., Knoxville, TN 37996, USA; 2 Center for Nanophase Materials Sciences, Oak Ridge National Laboratory, 1 Bethel Valley Rd., Oak Ridge, TN 37830, USA; 3 Materials Science and Technology Division, Oak Ridge National Laboratory, 1 Bethel Valley Rd., Oak Ridge, TN 37830, USA

**Keywords:** resistive switching, combinatorial sputtering, tantalum oxide, zirconium oxide, thin films

## Abstract

Combinatorial magnetron sputtering and electrical characterization were used to systematically study the impact of compositional changes in the resistive switching of transition metal oxides, specifically the Zr_x_Ta_1−x_O_y_ system. Current-voltage behavior across a range of temperatures provided insights into the mechanisms that contribute to differences in the electrical conductivity of the pristine Ta_2_O_5_ and ZrO_2_, and mixed Zr_x_Ta_1−x_O_y_ devices. The underlying conductive mechanism was found to be a mixture of charge trapping and ionic motion, where charge trapping/emission dictated the short-term cycling behavior while ion motion contributed to changes in the conduction with increased cycling number. ToF-SIMS was used to identify the origin of the “wake-up” behavior of the devices, revealing an ionic motion contribution. This understanding of how cation concentration affects conduction in mixed valence systems helps provide a foundation for a new approach toward manipulating resistive switching in these active layer materials.

## 1. Introduction

Amorphous oxides (TaO_x_, ZrO_x_) [1,2,3,4,5] and bulk phase change materials (VO_2_/V_2_O_5_) [6] are showing tremendous potential as active layer materials for resistive random access memory (ReRAM) materials. These oxides are compatible with existing complementary metal oxide semiconductor (CMOS) fabrication methods and have unique functional properties, such as the ability for in-memory analog computations, that could allow them to complement or replace conventional CMOS-based devices. For example, Ta_2_O_5_ has shown sub-nanosecond switching [7], low switching voltage (~1 V), long state retention (>10 years), and high switching endurance (>10^10^ cycles) [2]. Arrays of Pd/TaO_x_/Ta/Pd cross bar devices have demonstrated complex computational tasks with improved energy efficiency and throughput compared to digital systems [8]. The resistive switching mechanisms in these materials are driven by oxygen migration and typically require an initial electroforming step, wherein the film is switched from a high-resistance to a low-resistance state. This electroforming step is not fully understood and has not been thoroughly replicated in computational models [9], and is not always required for stable resistive switching [10]. However, it is generally considered important for initiating the onset of oxygen motion in highly insulating oxide films. This oxygen motion can be driven both in and out of the plane of the TaO_x_ layer, where the mechanism and the resulting resistive switching IV characteristics were shown by Lee et al. [2]. This mechanism shows a local movement of oxygen from the Ta_2_O_5-x_ layer into a TaO_2-x_ layer, forming metallic filaments through the Ta_2_O_5-x_ layer and a buildup of oxygen at the Ta_2_O_5-x_/TaO_2-x_ interface [2]. This process can be seen in the IV characteristics of the material and is typically seen as a sharp increase in current at the set voltage (V_set_) where the metallic filaments are formed. Lee et al. subsequently demonstrated the effect that the active layers [2] and electrode materials have on the device characteristics [3]. In situ transmission electron microscopy (TEM) was used to image the oxygen motion in the active layers and showed that, by decreasing the interface roughness between the top electrode and the Ta_2_O_5-x_ layer, the device behavior transitions from being dominated by filament formation to behavior that is dictated by the buildup of interfacial oxygen [3]. This suggests that rough interfaces can enable localization of the electric field and drive filament formation. The effects of silicon doping of TaO_x_ was studied by Kim et al., [11] where the doped devices showed higher on/off ratios, suggesting a larger depletion gap between the filament and electrode. This larger depletion gap was attributed to a higher oxygen mobility in the Si-doped tantalum oxide compared to the undoped oxide. This increase in oxygen motion was rationalized by the induction of oxygen mobility channels generated by stronger bonding in the Si-O bond relative to Ta-O.

Ta:ZrO systems, deposited by atomic layer deposition (ALD), have also been investigated by Kukli et al. [12], finding that the chemistry with a low zirconium concentration (Zr:Ta atomic ratio of 0.2) exhibited the largest hysteresis and most consistent switching. This chemistry showed the highest crystallization temperature (800 °C) forming a δ-Ta_2_O_5_ phase. Crystallization eliminated all hysteretic behavior for each chemistry studied, suggesting that the mechanism for resistive switching in these Zr_x_Ta_1−x_O_y_ chemistries requires vacancy-driven ion movement, which requires less energy in amorphous systems. Kukli et al. [12] showed that hysteresis is dependent on the Zr:Ta ratio and that this hysteresis requires an amorphous structure, suggesting that modulating the Zr:Ta ratio has an effect on the vacancy concentration of the oxide layer.

Combinatorial synthesis of Y:Zr_x_Ta_1−x_O_y_ by pulsed laser deposition (PLD) was conducted by Kiyota et al. [13]. The system consisted of a consistent 10 atomic % YO_x_ and a compositional gradient of Ta and Zr. A minimum in the dielectric constant (k) of 18 was found at 30% ZrO_2_ and then steadily increased to 22 with increasing Ta_2_O_5_. However, the leakage current also steadily increased with increasing Ta_2_O_5_. Zrinski et al. [14] investigated a combinatorial system of Ta_x_Hf_1−x_O_y_ deposited by magnetron sputtering and a subsequent wet electrochemical anodization process to oxidize the topmost 20 nm of the film. Current-voltage measurements revealed that the high-Hf concentration zone exhibited poor resistive switching behavior, while the low-Hf zone did not show any improvements compared to Ta_2_O_5_. Ratios between 50 and 70 atomic % Hf showed on/off ratios of eight orders of magnitude with device endurance reaching one million cycles. This region exhibited unipolar switching compared to the high and low Hf regions, which exhibited bipolar switching. This unipolar switching was driven by HfO_2_ crystallites present in the otherwise amorphous oxide.

Doolittle et al. observed resistive switching that was attributed to charge trapping in defect states in Nb_2_O_5-x_ films [15]. These Nb_2_O_5-x_ devices generated current-voltage hysteresis at low frequencies by increasing voltage over the onset voltage needed to transition from Schottky emission to tunneling transport through the highly charged interface layer of the film. This topmost layer causes the turn on voltage to be dictated by the Schottky barrier height. Beyond the turn-on voltage, the devices transition to a Fowler–Nordheim (FN) tunneling/Poole–Frenkel (PF) mechanism that dictates the hysteretic behavior. Kim et al. also produced charge trapping-based Nb_2_O_5-x_ memristors, but in order to stabilize the high and low-resistance states, Ta_2_O_5_ and Al_2_O_3-y_ capping layers were inserted between the Nb_2_O_5-x_ active layer and Pt and Ti electrodes, respectively [16]. The Ta_2_O_5_ layer prevented the discharge of energized trap states in the active layer to the Pt electrode, stabilizing the low-resistance state (LRS). The Al_2_O_3-y_ layer prevented charge migration from the Ti electrode to empty trap states in the active layer, thus stabilizing the high-resistance state (HRS). Similarly, electroforming-free hysteretic current-voltage behavior was shown by Yoon et al. [17] using a Pt/Ta_2_O_5_/HfO_2-x_/TiN device stack that employed ALD to deposit the Ta_2_O_5_ and HfO_2-x_ active layers.

Reservoir computing (RC) has emerged as a powerful and energy-efficient paradigm for processing time-dependent information by utilizing the dynamics of nonlinear electrical systems. Unlike traditional recurrent neural networks, RC fixes the internal reservoir weights and trains only the output layer, greatly simplifying the training process while maintaining high computational capability for tasks such as pattern recognition, classification, and temporal prediction. Recent advances have demonstrated that volatile memristive devices are excellent candidates for physical reservoir computing because of their intrinsic short-term memory behavior and nonlinear, history-dependent conductance modulation. Ju et al. implemented an 8-bit reservoir computing system using volatile ITO/ZrO_x_/TaN memristors, exploiting gradual interface-type resistive switching and current decay phenomena to achieve high-dimensional temporal mapping and efficient multi-level data storage [18]. A similar approach has also been reported using Pt/BFO/SRO/STO ferroelectric devices [19], highlighting the critical role of volatile resistive switching in constructing scalable, energy-efficient, and neuromorphic-compatible reservoir architectures.

This work leverages combinatorial thin-film sputtering to rapidly explore the ReRAM properties of mixed valence transition metal oxide active layers, where co-sputtering Ta and Zr metal targets in an Ar-O_2_ ambient generates a compositional gradient of mixed oxidation states. Chemical composition and valence state were correlated to local current-voltage measurements via X-ray photoelectron spectroscopy (XPS) and time-of-flight secondary ion mass spectrometry (ToF-SIMS) to elucidate the resistive switching mechanisms operative in the Zr_x_Ta_1−x_O_y_ system.

## 2. Materials and Methods

Plane-wave density functional theory (DFT) calculations were carried out using Vienna Ab initio Simulation Package (VASP), version 6.5, and the projector-augmented wave (PAW) pseudopotentials for electron–ion interactions [20]. Exchange–correlation interactions were accounted for using the generalized gradient approximation (GGA) functional of Perdew–Burke–Ernzerhof (PBE) [21]. A previous model was used for amorphous tantalum oxide. That model was based on a super cell of crystalline Ta_2_O_5_, containing 168 atoms (Ta_48_O_120_ [22,23]) with optimized lattice constants of *x* = 14.635 Å, *y* = 12.377 Å, and *z* = 11.761 Å. During structural optimization, the atoms and cell volume relaxed until the residual forces fell below 0.02 eV/Å, with a cutoff energy of 520 eV and Gamma-point k-point sampling. To generate an amorphous structure, ab-initio molecular dynamics (AIMD) simulations were used in a melt-and-quench approach. This model and its oxygen vacancy derivatives has shown very good agreement with experimental cathode luminescence (CL), electron energy loss spectroscopy (EELS), and scanning microwave impedance microscopy (SMIM) [24]. In this study, starting with amorphous Ta_48_O_119_, AIMD was run using a Nose–Hoover thermostat at room temperature and used the mean square displacement (MSD) of O via Einstein’s relation to find the respective diffusion coefficients. Following this simulation protocol, Zr was doped into the model following Zr_x_Ta_1−x_O_y;_ with x = 5, 15, 20 which corresponds to 10%, 31%, and 42% Zr doping. The largest MSD for oxygen and subsequently the highest diffusion coefficient was found to occur at a doping level of 31% Zr (see Supplemental Figure S1).

A 5 nm Ti adhesion layer was deposited using DC magnetron sputtering onto a thermally grown SiO_2_ (500 nm)-coated silicon substrate. The bottom 40 nm Pt electrode was subsequently deposited on the Ti adhesion layer via magnetron sputtering. Ta(20 nm)/Zr_x_Ta_1−x_O_y_(10–15 nm) active layers were deposited on top of the Pt(40 nm) bottom electrode. The metallic Ta layer was deposited with an applied power of 100 W DC for 1 min 50 s with substrate rotation on to realize a uniform film thickness. The combinatorial Zr_x_Ta_1−x_O_y_ layer was deposited with an applied power of 38 W DC on the Ta target and 100 W DC on the Zr target with a 25/5.2 sccm Ar/O_2_ flow rate ratio and 5 mTorr pressure for 5 min and 50 s with the substrate stationary to realize the combinatorial gradient. X-ray reflectometry (XRR) was used to measure the center thickness of the deposited layers (see Supplemental Figure S2) and energy dispersive spectroscopy (EDS) was used to verify the composition gradient of the film across the 100 mm substrate. Grazing incidence X-ray diffraction (GIXRD) was used to confirm the amorphous structure of the film (see Supplemental Figure S3). A PANalytical X’Pert Pro MRD equipped with a proportional Xenon counter was used for the XRR measurement and GIXRD, while a Zeiss Merlin SEM was used for the EDS measurement. XPS was also used to measure the composition and elucidate the bonding/valence characteristics in the films as the cation ratio varies across the sample. Films with pure Ta_2_O_5_ and ZrO_2_ were also fabricated using the same parameters but with substrate rotation on to reduce thickness gradients.

**Figure 1:**
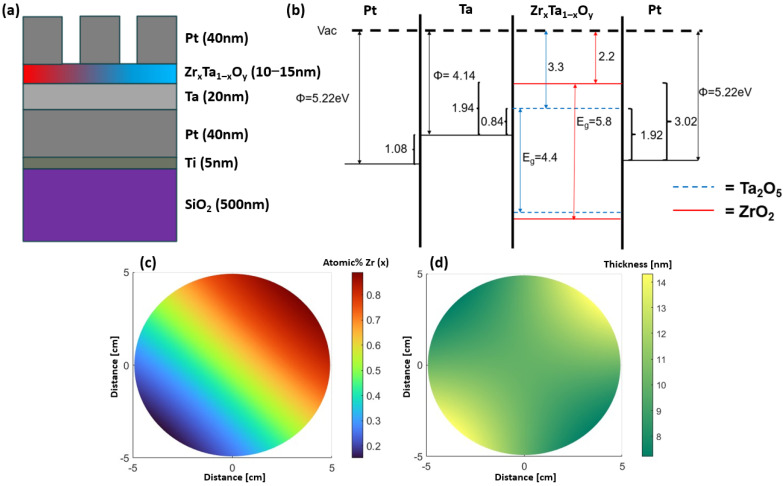
Schematic of the (**a**) thin film stack and (**b**) the expected band diagram of the devices. Schematic of (**c**) simulated cation atomic % Zr (note that atomic % Ta is 1−atomic % Zr), and (**d**) thickness of the combinatorial Zr_x_Ta_1−x_O_y_ layer. The ZrO_2_ bandgap was taken from Li et al. [26], Ta_2_O_5_ bandgap from Zhuo et al. [27], while Pt and Ta work functions were taken from Mechtly [28].

Then, 100 mm diameter, 40 nm thick top Pt electrode pads were DC magnetron sputter-deposited via a photolithographically patterned lift-off process. Electrical cycling measurements were performed by biasing the Pt top electrodes with a Keithley 2400 source measure unit (SMU) while the continuous bottom Pt electrode was grounded. The electrical measurements were conducted along the Zr_x_Ta_1−x_O_y_ composition gradient at various electrode positions representative of various stoichiometries. Each DC electrical cycle had a constant 1 min cycle time and consisted of 400 voltage increments where the voltage increment varied based on the voltage range studied. The leakage current behavior over time was measured using a Keithley 4200A-SCS parameter analyzer, manufactured by Tektronix (Beaverton, OR, USA). All electrical measurements utilized a Form Factor Cascade CM300xi probe station. A diagram of the film stack along with the corresponding band diagram is illustrated in Figure 1. Also shown in Figure 1 is the estimated cation composition gradient and thickness across the 100 mm diameter substrate.

Time-of-flight secondary ion mass spectrometry (ToF-SIMS) system, ToF.SIMS 5 by *iONTOF* (Münster, Germany), was used to profile pristine and cycled devices. An alternating sputter of Cs^+^ and Bi_3_^+^ was used to profile and image the device. A 1 keV Cs^+^ ion beam was rastered in a 500 × 500 µm^2^ area to form a sputtered crater for 20 frames. Four frames of a 30 keV Bi_3_^+^ ion beam were used to raster a 100 × 100 µm^2^ area. An electron flood gun was used to mitigate charging during profiling. Figure S4a,c,e,f show images of the CsPt^+^ ion count in order to show the relative position of the device centered in the ion beam raster. Figure S4b,d,f,h show the areas of interest that were used for the depth profiles to isolate ions from the device and exclude ions from the native film in the corners of the 100 × 100 µm^2^ scan.

## 3. Results and Discussion

The device film stack geometry can be found below in Figure 1a, alongside the theoretical band diagram (Figure 1b). The band diagram of the film stack illustrates the large barrier to charge injection from the top Pt electrode as compared to injection from the bottom Pt/Ta electrode. The predicted cation concentration (Figure 1c) and thickness (Figure 1d) of the memristive oxide layers were simulated across the wafer; many of the parameters that are relevant for the sputtering system were experimentally determined in the previous work [25].

Figure S2 shows a XRR thickness measurement at the center of the combinatorial wafer while Figure S5 compares the measured (EDS) and simulated [25] Ta and Zr cation atomic percentage measured along the centerline of the substrate, which are in good agreement. For the results presented below, the cation compositions were estimated from the simulated sputtering model. Calculated mean square displacement (MSD) values were determined at various Ta/Zr ratios and showed an increase in oxygen mobility around 30% Zr. The MSD plot of oxygen for three different Zr doping concentrations is shown in Figure S1.

**Figure 2:**
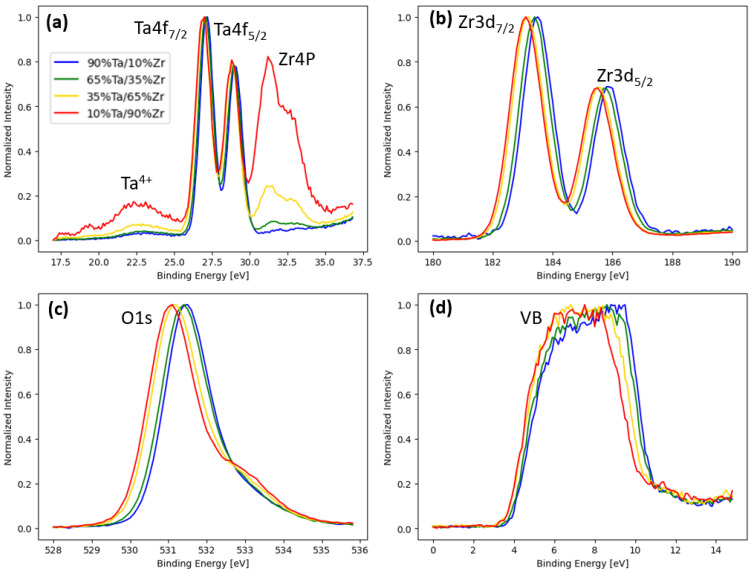
Comparison of normalized binding energy for (**a**) the Ta 4f peaks, (**b**) the Zr 3d peaks, (**c**) the O 1s peak, and (**d**) valence band across the combinatorial Zr_x_Ta_1−x_O_y_ film.

Figure 2a–d are XPS spectra for the Ta 4f (and Zr 4p), Ta 3d, O 1s, and the valence band energy regions, respectively. As can be observed, all of the spectra shift to lower binding energy with increasing Zr content. The shifts in the Ta, Zr, and O core levels (b–d) are consistent with observations of Tewg et al. that were attributed to charge transfer mechanisms affecting all three elements in the film [29]. Namely, as Zr concentration is increased, the positive charge of the cation Ta and Zr decreases and the negative anion charge on the oxygen increases. As noted in the Ta 4f spectra, the Ta^4+^ peak increases with increasing Zr, which affirms the decrease in the overall charge on the Ta cations. Interestingly, the valence band also shifts to lower binding energy with increasing Zr content, which is not consistent with the band diagram pictured in Figure 1. This suggests that perhaps the electron affinity of the Ta_2_O_5_ is slightly larger or the ZrO_2_ is slightly smaller than the diagram suggests; in either case, the conduction band barrier height difference between the ZrO_2_ and Ta_2_O_5_ would be larger than the 1.1 eV represented in Figure 1.

**Figure 3:**
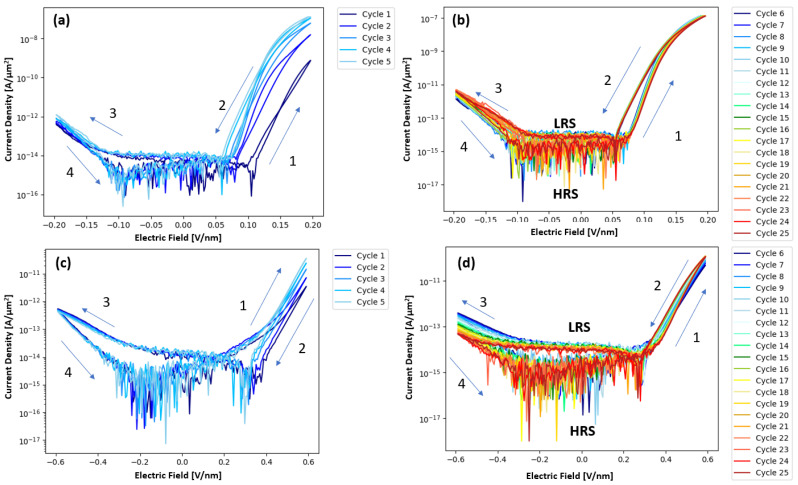
Current density vs. electric field characteristics of a Pt/Ta/Ta_2_O_5_/Ta device for (**a**) first 5 cycles and (**b**) cycles 6–25. Current density electric field behavior of Pt/Ta/ZrO_2_/Pt device for (**c**) the first 5 cycles illustrating the wake-up cycles, and (**d**) cycles 6–25 where smaller shifts are observed. HRS and LRS signify the low-resistance state and high-resistance state, respectively. Note that the arrows in the figures show the order of the cycling.

**Figure 4:**
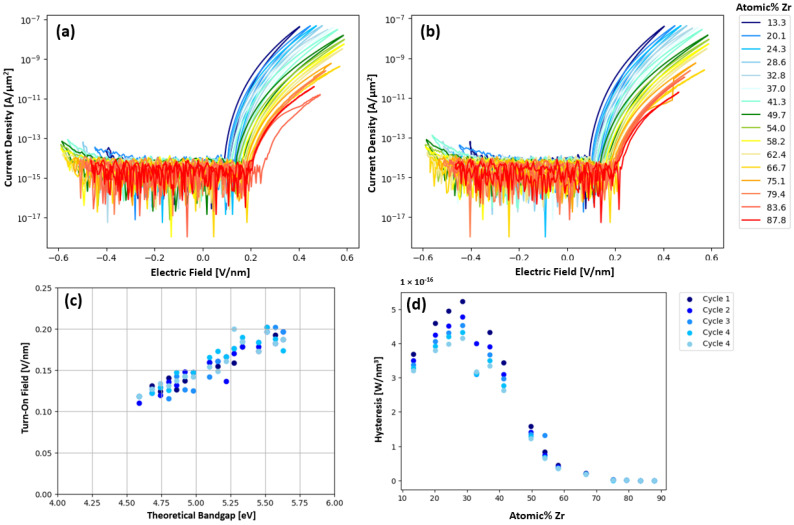
Current density versus electric field for (**a**) cycle 1 and (**b**) cycle 5 as a function of composition across the combinatorial Zr_x_Ta_1−x_O_y_ wafer. The relationship of (**c**) turn-on field and theoretical bandgap (composition), and (**d**) hysteresis versus Zr concentration.

Figure 3a,b illustrate the current density electric field measurements of the pure Ta_2_O_5_ devices, which revealed a “wake-up” behavior of about five cycles at +/− 0.2 V/nm. After these wakeup cycles, cycles 6–25 showed consistent resistive switching where the devices switch from the HRS to the LRS upon the forward and reverse positive voltage cycle. The post wake-up behavior of both devices shows a distinct turn-on voltage. Arrangement of the probes indicates that the positive voltage cycle is due to electron injection from the Ta interlayer bottom electrode of the device, which is consistent with the lower work function and barrier height for the Ta/Ta_2_O_5_ interface. The negative voltage sweep for injection from the Pt/Ta_2_O_5_ top electrode has a much higher barrier height and consequently higher turn-on field and, thus, rectifying behavior. In contrast to the Ta_2_O_5_ devices, Figure 3c,d show that the pure ZrO_2_ devices exhibit a longer wake up period of about 15 cycles, after which the ZrO_2_ devices also exhibit resistive switching from HRS to LRS. Plots showing cycles 6–10, 11–15, 16–20, and 21–25 for a pristine ZrO_2_ device can be seen in Figure S6. During the initial cycling, the ZrO_2_ devices exhibit switching from an LRS to an HRS, before slowly transitioning to high-to-low resistance state switching. This transition from LRS/HRS switching to HRS/LRS switching is attributed to a shift from charge capture-controlled PF conduction, where charge capture rate is above the charge emission rate, to emission-controlled PF conduction. This shift is caused by a decrease in trap depth with increased cycling, effectively increasing the charge emission rate. The ZrO_2_ devices also show decreasing current (higher resistance) during subsequent negative sweeps and this negative polarity switching did not saturate up to 25 sweeps. When measuring Zr_x_Ta_1−x_O_y_ combinatorial devices, the wakeup period is not as pronounced compared to the pure Ta_2_O_5_ or ZrO_2_ devices. The current density versus electric field for Zr_x_Ta_1−x_O_y_ is plotted as a function of the composition (normalized by the thickness gradient from Figure 1d in Figure 4a,b for cycle 1 and 5, respectively, where a composition range of 0.133 < x < 0.878 was measured. The turn-on field was defined as the field where the current begins to increase above the low voltage noise. Figure 4c shows the expected trend, that the turn-on field increases linearly with the theoretical bandgap. The theoretical bandgap was calculated assuming the bandgap scales linearly with Zr content. Figure 4d shows that hysteresis increases up to about 30% Zr then steadily decreases with increasing Zr. This maximum hysteresis at 30% Zr matches the concentration (30% Zr) found by Kiyota et al. to have the minimum dielectric constant in a Y:Zr_x_Ta_1−x_O_y_ system [13]. This maximum hysteresis concentration is also closest to the concentration (Zr:Ta atomic ratio of 0.2) found by Kukli et al. [12] to have the largest hysteresis and most consistent switching behavior. The AIMD simulations presented in Figure S1 also show the highest MSD for oxygen at a Zr concentration of 30 atomic percent. This result suggests that ionic motion could also be contributing to the increased hysteresis around 30% Zr region.

To understand the mechanisms responsible for the hysteresis observed in the Zr_x_Ta_1−x_O_y_ system, various current injection mechanisms that can be operative in metal–insulator–metal (MIM) devices were explored. The current density electric field behavior is similar to what was observed by Doolittle et al. for memristive Nb_2_O_5_ devices, where Schottky emission was determined to be the primary conduction mechanism prior to the turn-on voltage [15]. This is supported by Figure 4c, showing the linear increase in the turn-on field with increasing zirconium content. After current onset, trap-assisted Fowler–Nordheim (FN) tunneling dominates current injection through the highly charged layer at the electrode/Zr_x_Ta_1−x_O_y_ interface, while Poole–Frankel (PF) charge trapping and emission is responsible for conduction through the remainder of the amorphous Zr_x_Ta_1−x_O_y_ film. During the reverse sweeps, hysteresis occurs because the emission rate is higher than the charge capture rate, which allows for higher conductivity in the reverse sweep relative to the forward sweep. It is also suspected that a portion of the trap states remain filled during the reverse sweep, effectively increasing the charge emission/capture ratio. This current limited electron capture process is dependent on the emission rate by trap states (*e_e_*),

(1) $$e_{e}=v_{th}\sigma_{n}N_{C}N_{t}exp\left(-\frac{E_{C}-E_{t}}{k_{B}T}\right),$$

and charge capture rates (*c_e_*),

(2) $$c_{e}=v_{th}\sigma_{n}nN_{t},$$

where *v_th_* is the carrier thermal velocity, *σ_n_* is the electron capture cross section, *n* is the density of available electrons, *N_t_* is density of traps, *N_c_* is the effective density of states and *E_c_*−*E_t_* is the trap depth. In this case, the primary charge carrier for this mechanism is electron motion.

**Figure 5:**
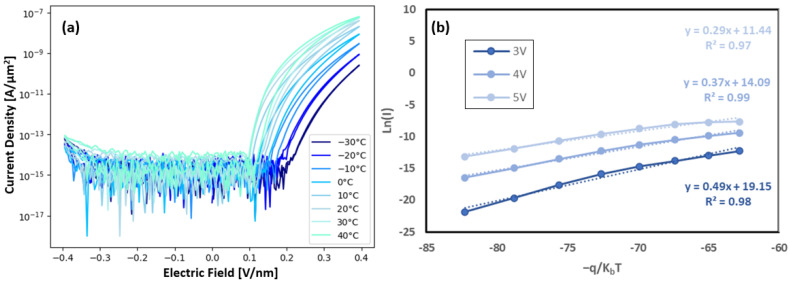
Electronic behavior of Zr_3_Ta_7_O_y_ device at various temperatures. Shows (**a**) current density and electric field relationship and (**b**) Ln(I) versus −q/KbT plot for various voltages which are used to determine the PF activation energy (slope) for each temperature.

This temperature-driven inequality of emission and capture of charge is confirmed in Figure 5a, where measurements were conducted across a range of temperatures and show that the calculated hysteresis (Figure S7a) increases with increasing temperature for the five cycles measured. An increase in the maximum current density with temperature is demonstrated in Figure 5a, which could be explained by an increase in thermal carrier velocity and the number of trap states. This increase in current and hysteresis with temperature also suggests a possible ionic motion contribution to the conduction mechanism. A similar combination of Schottky emission at low voltages and tunneling/PF conduction at higher voltages was observed by Doolittle et al. [15]. PF conduction is observed at voltages above the turn on voltage while Schottky emission is driven by a highly charged layer at the electrode/active layer interface. Poole–Frankel conduction is described by

(3) $$J_{PF}=qµN_{0}E\;exp\left(-\frac{q}{K_{B}T}\left(\Phi_{D}-\sqrt{\frac{qE}{\pi \varepsilon }}\right)\right)$$

where *q* is elemental charge, *µ* is electron mobility, *N*_0_ is defect concentration, *E* is electric field, *Φ_D_* is defect trap energy, *ε* is the high-frequency dielectric constant. As shown in Figure S7b, the defect trap energy (*Φ_D_*) of a device with Zr_3_Ta_7_O_y_ was determined to be 1.16 eV from the intercept of a plot of the activation energy versus the square root of electric field (Figure 5b). This trap energy is significantly higher compared to the trap energy found by Doolittle et al. in Nb_2_O_5_ (0.22 eV) [15]. These values for Nb_2_O_5_ and Zr_x_Ta_1−x_O_y_ are qualitatively consistent considering Zr_x_Ta_1−x_O_y_ exhibited a higher onset field (0.125 V/nm versus 0.005–0.09 V/nm in Nb_2_O_5_). The negative bias portion of the IV cycling shows significantly lower current due to a higher Schottky barrier height at the Pt/oxide interface.

Similar plots for the Ta_2_O_5_ devices are shown in Figure S8. The Ta_2_O_5_ injection shows a decrease in PF trap activation energy with increasing field with a zero-field trap depth of 0.93 eV. The increased trap depth of the Zr_3_Ta_7_O_y_ film compared to the pure Ta_2_O_5_ and ZrO_2_ films is attributed to the increasing bandgap with the addition of ZrO_2_ to Ta_2_O_5_ and thus deeper trap states. The increased trap depth also suggests that the conduction mechanism of the mixed Ta/Zr cation film exhibits a similar conduction mechanism to the Ta_2_O_5_ devices, at least at modest amounts of Zr. Figure S9 shows the current voltage behavior for the ZrO_2_ injection along with the PF activation energy and trap depth calculations. Counter to the Ta_2_O_5_ injection, the ZrO_2_ devices show an increasing PF trap activation energy with increasing field, generating a zero-field trap depth of 0.05 eV. The low trap depth of the ZrO_2_ devices suggests that the PF trap-emission mechanism is not the dominate current transport mechanism in the pure ZrO_2_ film.

**Figure 6:**
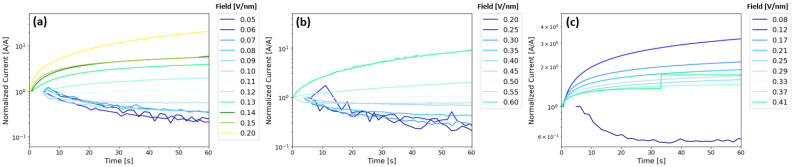
Normalized leakage current versus time for (**a**) Ta_2_O_5_ devices (**b**), ZrO_2_ devices, and (**c**) Zr_3_Ta_7_O_y_ devices at various electric fields.

Following Doolittle et al., Figure 6 shows normalized current versus time as a function of bias voltage for Ta_2_O_5_ (Figure 6a), ZrO_2_ (Figure 6b), and Zr_3_Ta_7_O_y_ (Figure 6c), all poled such that electron injection is from the bottom Pt/Ta electrode (or positive voltage applied to the top Pt contact). See Figures S10 and S11 for the corresponding absolute values of the current-time plots and initial current density values versus field for each active layer, respectively. The Ta_2_O_5_ injection shows a decrease in current versus time below the turn-on field (~0.11 V/nm) and a current increase above the turn-on field. This signature is consistent with a transition in the mechanism from Schottky emission below the turn-on field to trap-assisted tunneling and Poole–Frenkel trap hopping above the turn-on field. Similarly, the ZrO_2_ device exhibits a shift from decreasing current versus time to increasing current versus time at the turn-on field around ~0.5 V/nm. The higher turn-on field is due to an increase in barrier height of the Ta/ZrO_2_ interface. While the exact current transport mechanism above turn-on is not clear due to the temperature-dependent behavior observed in Figure S9, it is interesting that the device also demonstrates a similar change in the current versus time below and above the turn-on field. The Zr_3_Ta_7_O_y_ device also demonstrated a rise in current above the turn-on field, similar to the Ta_2_O_5_ and ZrO_2_ devices, and has a threshold in the behavior between 0.08 and 0.12 V/nm. While the rise in the normalized current in the Zr_3_Ta_7_O_y_ device above the turn-on field decreases in magnitude with increasing field, the absolute values of current are higher (see Figure S10). Also note that the Zr_3_Ta_7_O_y_ device broke down (and reached 1 mA compliance) after 30 s at a field of 0.41 V/nm.

**Figure 7:**
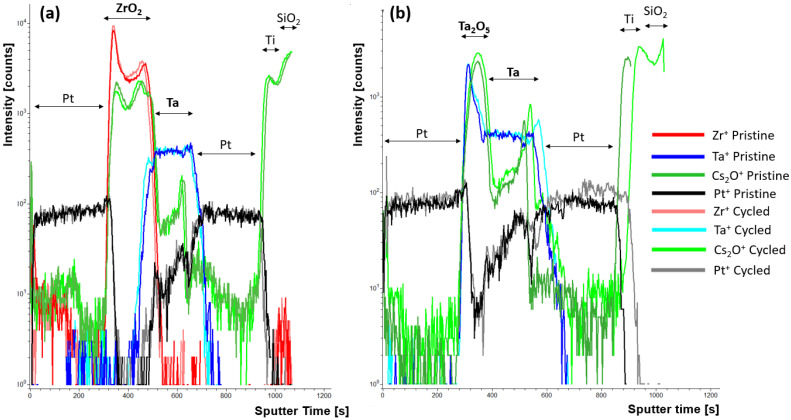
Ta^+^, Zr^+^, CsO_2_^+^, and Pt^+^ signals of pristine and cycled (**a**) ZrO_2_ devices and (**b**) Ta_2_O_5_ devices. The labels indicating the device active layers are in bold font. The profiles were aligned to the top Pt/oxide interface to account for any ion current or thickness variations in the surface or top Pt electrode layers.

While the mechanisms proposed for the observed hysteresis are attributed to the PF conduction and trap-assisted tunneling, to elucidate whether ionic motion was participating in the observed wake-up behavior observed in the ZrO_2_ and Ta_2_O_5_ devices, TOF-SIMS depth profiles were performed on pristine devices and compared to devices after 25 measurement cycles. The ToF-SIMS depth profiles, shown in Figure 7a, show shifts in Cs_2_O^+^ (surrogate for oxygen), Ta^+^ and Zr^+^ signals at the top Pt/ZrO_2_ and Ta/ZrO_2_ interfaces after cycling the device 25×. This suggests that there is some oxygen motion within the active layer during this wake-up period. The corresponding current-voltage cycling can be found in Figure S12. The relevant signals normalized to the total signal are shown in Figures S13 and S14 for the ZrO_2_ and Ta_2_O_5_ devices, respectively. In the ZrO_2_ device, oxygen can be seen to migrate in the ZrO_2_ layer toward the bottom Ta layer. This migration is tracked using the Cs_2_O^+^ signal. As Doolittle et al. [15] suggests, a highly charged layer generated at the electrode/active layer interface can result in increased tunnel injection during the during the positive sweep. Thus, the observed oxygen buildup observed at the ZrO_2_/Ta interface could be inducing a highly charged layer or oxidizing the interface and thus locally increasing the electric field, which is consistent with the observed maximum current increase observed with increasing cycle number during positive poling. Tracking the Zr^+^ signal shows a concomitant migration of Zr^+^ at the Ta interface. This could be due to slight densification during the wake-up or demixing at the ZrO_2_/Ta interface. In the Ta_2_O_5_ devices shown in Figure 7b, minimal change is observed in the Cs_2_O^+^ signal and the Ta^+^ signal. This is likely due to the smaller fields required to operate and wake the device up. The spike in Ta^+^ count at the Pt/Ta_2_O_5_ interface is attributed to the matrix effect generating increased ionization rates at interfaces due to variation in bonding compared to the bulk of the film [30,31]. It is interesting that there is a slight expansion of the Ta layer observed, along with a buildup of oxygen bottom Ta/Pt interface (though note that the scale is logarithmic, so a low concentration relative the Ta_2_O_5_ layer concentration). These changes at the Ta/Pt interface are not expected to substantially change the conduction mechanism in the Ta_2_O_5_ devices as a majority of the voltage drop should be across the Ta_2_O_5_ layer. The shift in Pt^+^ at in the Ta_2_O_5_ device is attributed to thickness variation across the sample in the bottom Pt layer and is not expected to affect device performance.

## 4. Conclusions

A combinatorial thin film synthesis process was used to rapidly investigate the memristive properties of a Zr_x_Ta_1−x_O_y_ system. Varying the Zr concentration systematically increased the bandgap while also increasing the trap depth due to the formation of more complex defect states. The underlying conductive mechanism of the Zr_x_Ta_1−x_O_y_ was found to be a combination of charge trapping and ionic motion, where charge trapping/emission dictated the short-term cycling behavior while, over time, ion motion contributed to changes in the conduction with increased cycling number. EDS was used to characterize the composition gradient and device characteristics were measured as a function of the cation concentration. XPS showed that all of the core level Ta 4f, Zr 3d, and O 1s spectra shift to lower binding energy with increasing Zr content, consistent with energy transfer among the ions. The Zr_x_Ta_1−x_O_y_ system showed volatile resistive switching, consistent with PF charge trapping behavior, induced by a transition from Schottky emission to trap-assisted tunneling through the highly charged layer at the electrode/active layer interface. The devices exhibited hysteretic diode behavior with a defined turn-on voltage and two distinct resistance states. The hysteresis area peaks around 30% Zr (relative to the total cation concentration of Ta + Zr = 100%), which corelates well with ab initio molecular dynamics (AIMD) simulations which showed maximum mobility at ~31% Zr doping. An increase in current and hysteresis with increasing temperature suggests an increase in thermal carrier velocity and trap states along with a possible ionic motion component. Trap activation energy was highest for the Zr_3_Ta_7_O_y_ active layer (1.16 eV) since the mixed valences of Ta^5+^ and Zr^4+^ generate deeper trap states compared to pure Ta_2_O_5_ and ZrO_2_. Slight ionic motion was observed in the ToF-SIMS depth profiles of cycled compared to pristine ZrO_2_ and Ta_2_O_5_ devices. In the ZrO_2_ devices, the ZrO_2_ layer width decreased with increased cycling along. The Ta_2_O_5_ devices only showed an increase in the Ta layer thickness after cycling, along with a buildup of oxygen at the bottom Ta/Pt interface. Future studies will work toward increasing the long-term stability and increasing the HRS/LRS ratio of the devices to elucidate the temporal charging/discharging response of the non-volatile device.

## Data Availability

The raw data supporting the conclusions of this article will be made available by the authors on request.

## Supplementary Materials

The following supporting information can be downloaded at: https://www.mdpi.com/article/10.3390/nano15100732/s1. Figure S1: Mean square displacement of oxygen for 10%, 31%, and 40% Zr doped tantalum; Figure S2: Shows XRR results of a measurement taken in the middle of the Zr_x_Ta_1−x_O_y_ combinatorial wafer; Figure S3: Grazing incidence x-ray diffraction (GIXRD) of the combinatorial film confirming the amorphous structure. The measurements at 86.1%, 69.2%, 52.3% and 35.3% Zr were offset by +400, +300, +200 and +100, respectively; Figure S4: Shows CsPt^+^ signal of the pristine Ta_2_O_5_ device (a) and the area of interest used for the depth profiles (b) in order to isolate the ion signal from the devices and exclude the native film on the corners of the 100 micron scan. Similar CsPt^+^ signal images and the corresponding region of interest are shown for the cycled Ta_2_O_5_ device (c,d), the pristine ZrO_2_ device (e,f) and the cycled ZrO_2_ device (g,h), respectively. These images correspond to the dataset shown in Figure 7; Figure S5: Shows comparison of chemistry model compared to EDS measurements across the Zr_x_Ta_1-x_O_y_ film; Figure S6: Shows current density and electric field relationship of ZrO_2_ device before (a,b) and after (c,d) positive polarity is stabilized.; Figure S7: Shows hysteresis (a) and activation energy (b) curves for pad a Zr_3_Ta_7_O_y_ device. Note the hysteresis increases of the for each cycle number as a function of increasing temperature a). (b) shows the plot of the activation energy versus the square root of electric field, where the y-intercept is the PF trap energy depth; Figure S8: Current-Voltage behavior (a), PF activation energy (b) and trap depth (c) for Ta_2_O_5_ devices; Figure S9: Current-Voltage behavior (a), PF activation energy (b) and trap depth (c) for ZrO_2_ devices; Figure S10: Leakage current for ZrO_2_ devices (a), Ta_2_O_5_ devices (b) and Zr_3_Ta_7_O_y_ devices (c) at various voltages over time. Figures (d–f) show current density vs time at the equivalent electric fields to Figures (a–c), respectively; Figure S11: Initial current density of leakage current measurements for Ta_2_O_5_, ZrO_2_ and Zr_3_Ta_7_O_y_ devices; Figure S12: Current-voltage cycling that corresponds to cycled SIMS devices. ZrO_2_ devices cycled 25x, (a) and (c), and Ta2O_5_ devices cycled 25x, (b) and (d). Ta_2_O_5_ cycled device was 9mm away from pristine Ta_2_O_5_ device while ZrO_2_ cycled device was 1mm away from pristine ZrO_2_ device. The increased noise in these cycles compared to Figure 3 is attributed to the addition of silver paint to the circuit that, that was used in order to ground the device; Figure S13: Shows the Cs_2_O^+^ (a), Zr^+^ (b) and Ta^+^ (c) normalized to the total signal. Corresponds to Figure 7a; Figure S14: Shows Cs_2_O^+^ (a) and Ta^+^ (b) signals normalized to the total signal. Corresponds to Figure 7b.
