# Supplementary material for: Electrical Behavior of Combinatorial Thin-Film ZrxTa1−xOy
Source: Nanomaterials (Basel). 2025 May 14;15(10):732. doi: 10.3390/nano15100732 (PMC12114200; doi:10.3390/nano15100732)
Supplement: Supplementary file 1 [file nanomaterials-15-00732-s001.zip › nanomaterials-3607334-supplementary.pdf]

nanomaterials

Electrical Behavior of Combinatorial Thin-Film $Zr_xTa_{1-x}O_y$

Matthew Flynn-Hepford [1], Reece Emery [1], Steven J. Randolph [2], Scott T. Retterer [2], Gyula Eres [3], Bobby G. Sumpter [2], Anton V. Ievlev [2], Olga S. Ovchinnikova [1] and Philip D. Rack [1,*]

[1] Materials Science & Engineering, University of Tennessee, 1505 Middle Dr., Knoxville, TN; mflynnhe@vols.utk.edu (M.F.-H.);remery1@vols.utk.edu (R.E.); oovchinn@utk.edu (O.S.O.),
[2] Center for Nanophase Materials Sciences, Oak Ridge National Laboratory, 1 Bethel Valley Rd, Oak Ridge, TN, 37830, USA; randolphsj@ornl.gov (S.J.R.); rettererst@ornl.gov (S.T.R.); sumpterbg@ornl.gov (B.G.S.); ievlevav@ornl.gov (A.V.I.)
[3] Materials Science and Technology Division, Oak Ridge National Laboratory, 1 Bethel Valley Rd, Oak Ridge, TN, 37830, USA; eresg@ornl.gov (G. E.)
* Correspondence: prack@utk.edu

Supplemental Information

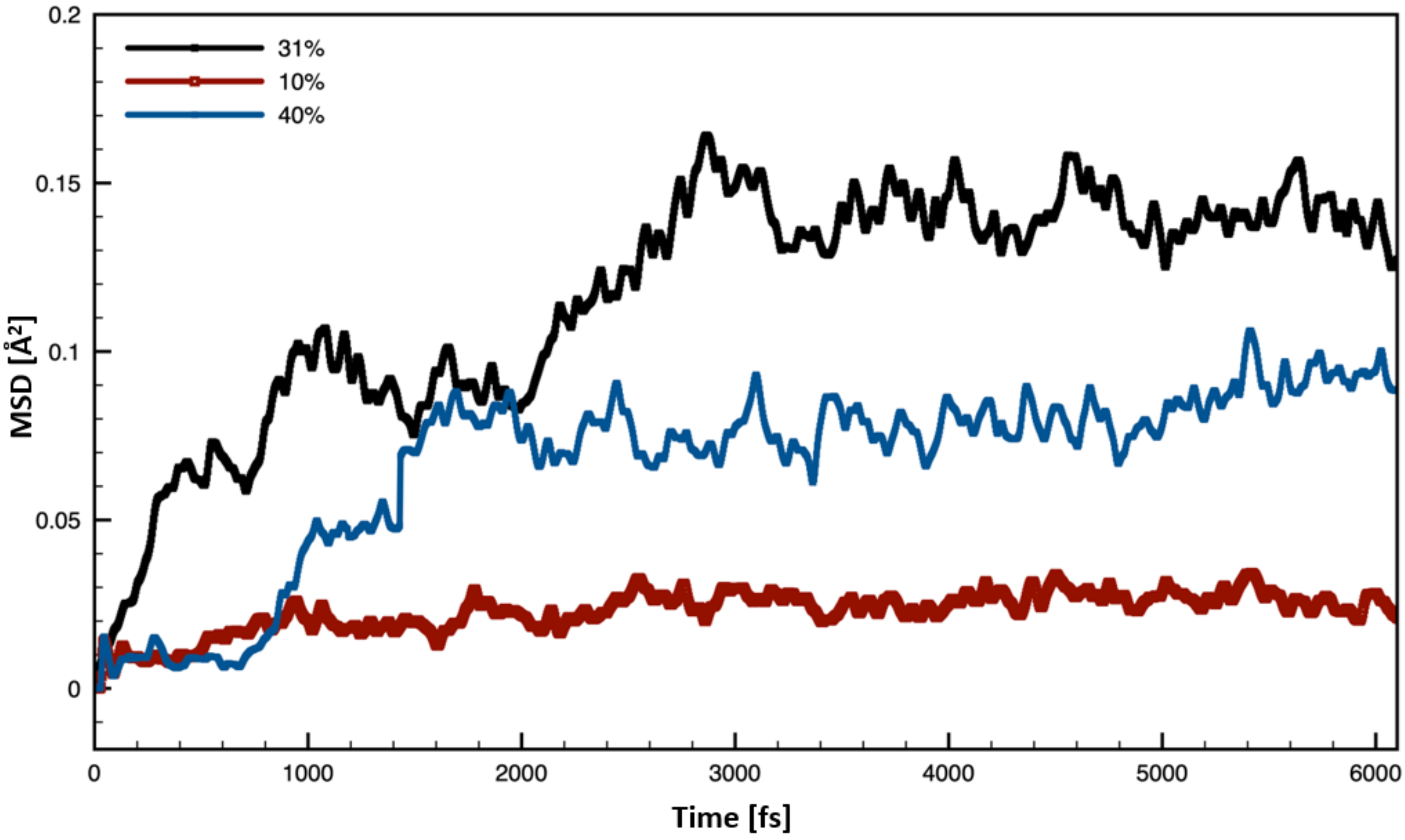

Figure S1. Mean square displacement of oxygen for 10%, 31%, and 40% Zr doped tantalum.

XRR results (measured prior to adding Pt caps)

Layer	Layer Description	Density (g/cm3)	Thickness (nm)	Roughness (nm)	Delta, e-7	Beta, e-7
5, 0	ZincBlende, Ta2O50.500 ZrO0.500	9	10.349	1.342	232.47339	14.91413
4, 0	DensityOnly, Ta	10.077	20.01	0.861	237.93819	20.50842
3, 0	DensityOnly, Pt	22.781	42.797	0.398	544.69691	55.85802
2, 0	DensityOnly, Ti	4.101	4.928	0.599	121.76695	10.4566
1, 0	DensityOnly, SiO2	2.64	513.079	0.017	85.44901	1.11502
Substrate	Diamond, Si	2.328	600000	0.139	75.68502	1.72955

Blue= data, Red = model fit.

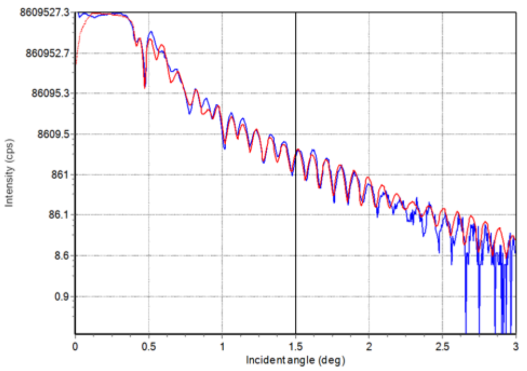

Figure S2. Shows XRR results of a measurement taken in the middle of the $Zr_xTa_{1-x}O_y$ combinatorial wafer.

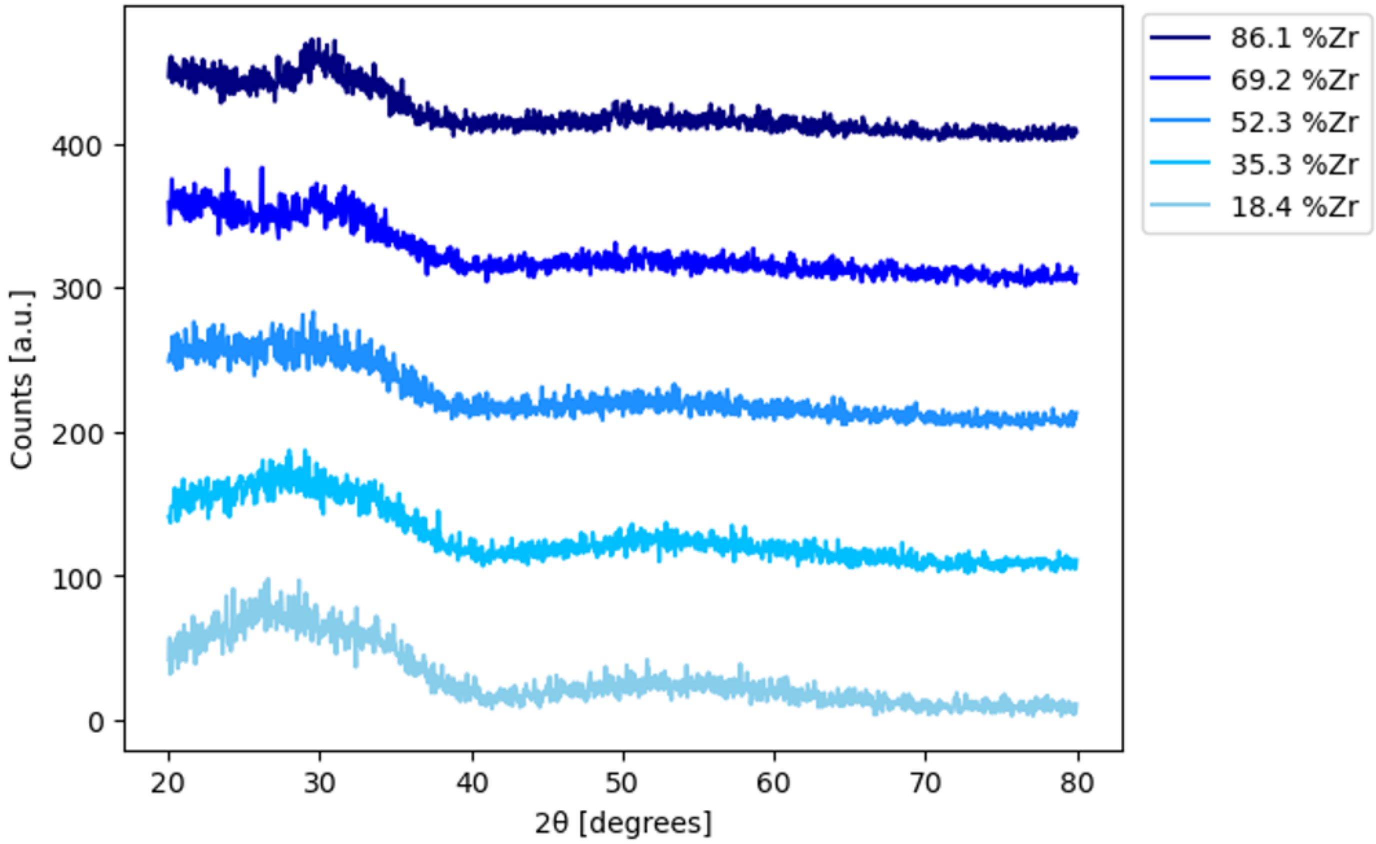

Figure S3. Grazing incidence x-ray diffraction (GIXRD) of the combinatorial film confirming the amorphous structure. The measurements at 86.1%, 69.2%, 52.3% and 35.3% Zr were offset by +400, +300, +200 and +100, respectively.

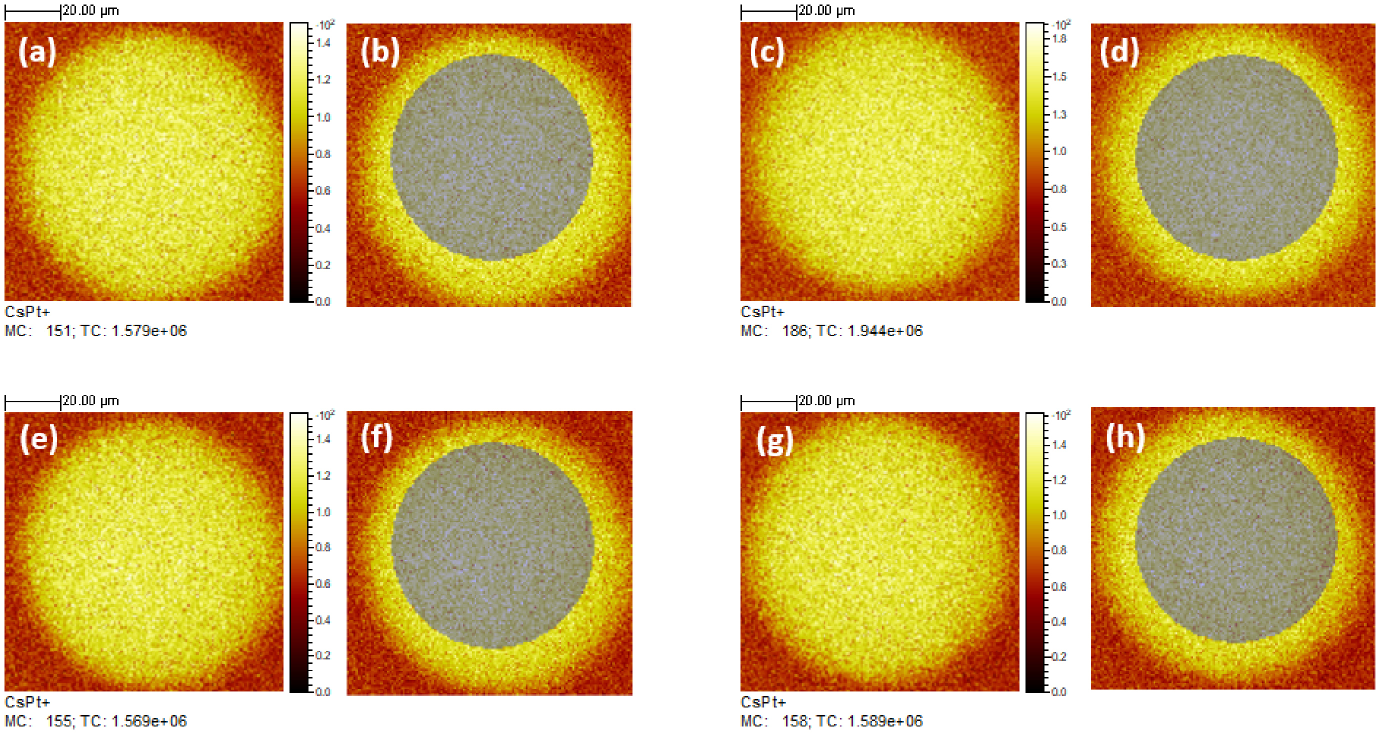

Figure S4. Shows $CsPt^+$ signal of the pristine Ta_2O_5 device (a) and the area of interest used for the depth profiles (b) in order to isolate the ion signal from the devices and exclude the native film on the corners of the 100 micron scan. Similar $CsPt^+$ signal images and the corresponding region of interest are shown for the cycled Ta_2O_5 device (c,d), the pristine ZrO_2 device (e,f) and the cycled ZrO_2 device (g, h), respectively. These images correspond to the dataset shown in Figure 7.

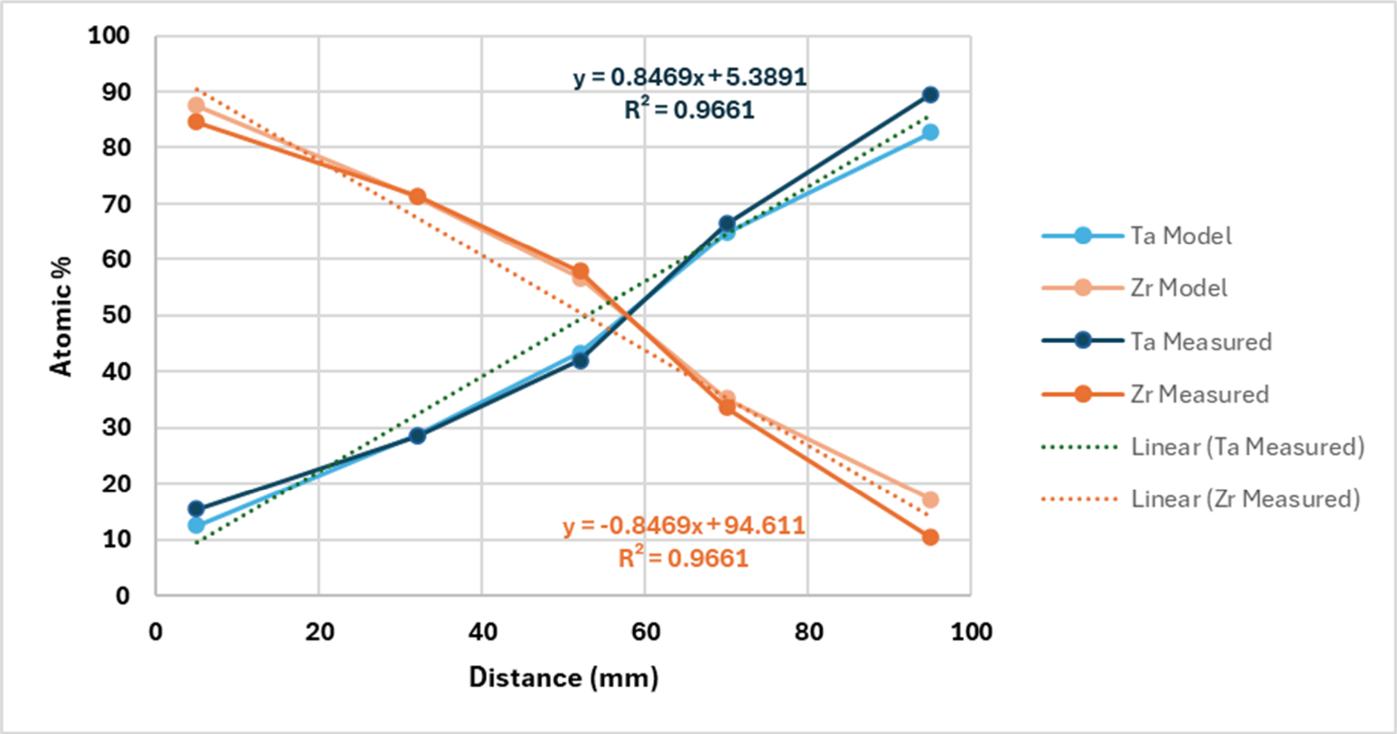

Figure S5. Shows comparison of chemistry model compared to EDS measurements across the $Zr_xTa_{1-x}O_y$ film

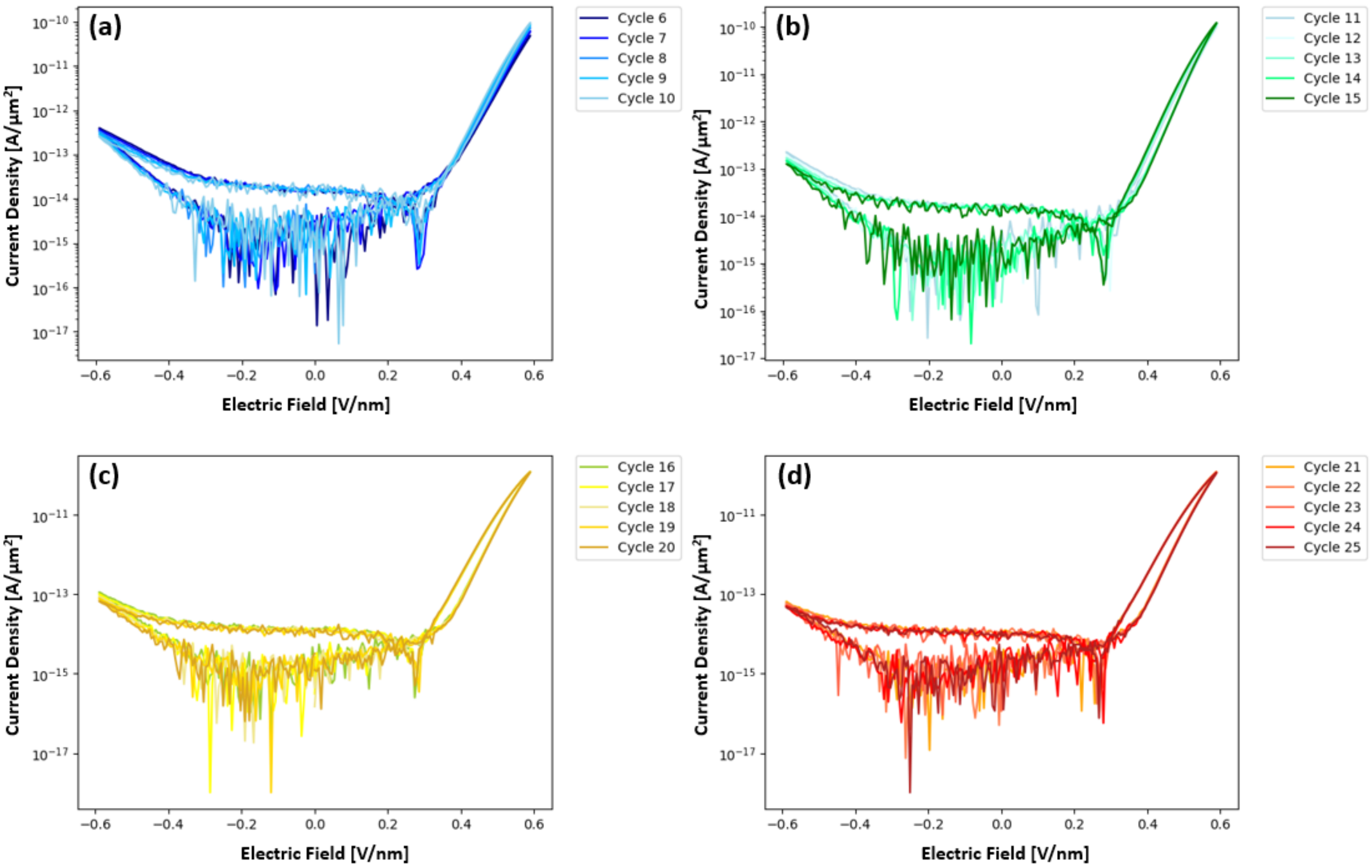

Figure S6. Shows current density and electric field relationship of ZrO_2 device before (a-b) and after (c-d) positive polarity is stabilized.

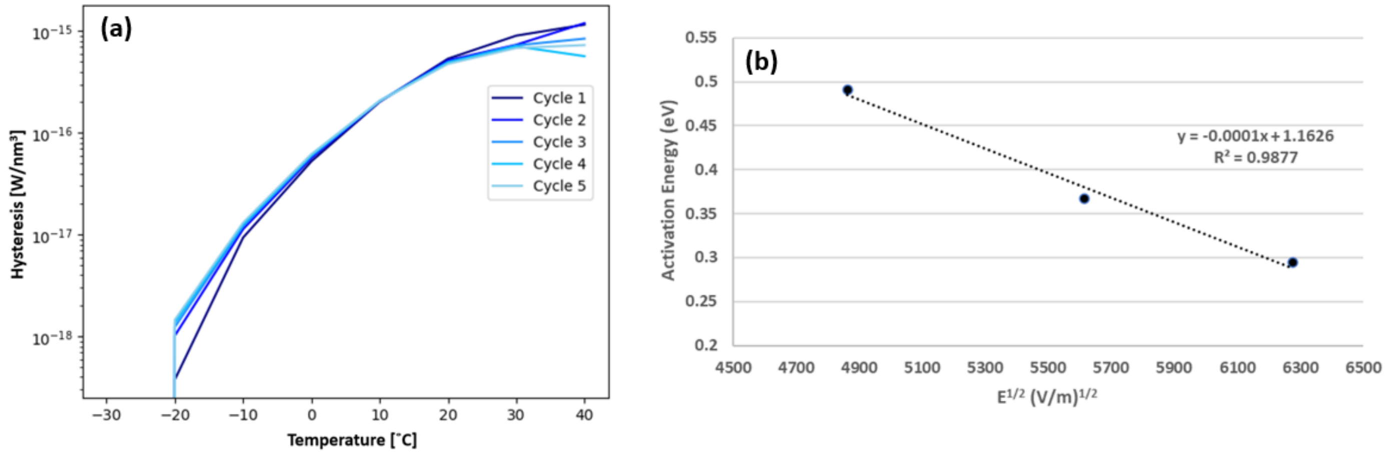

Figure S7. Shows hysteresis (a) and activation energy (b) curves for a $Zr_3Ta_7O_y$ device. Note the hysteresis increases of the for each cycle number as a function of increasing temperature a). (b) shows the plot of the activation energy versus the square root of electric field, where the y-intercept is the PF trap energy depth.

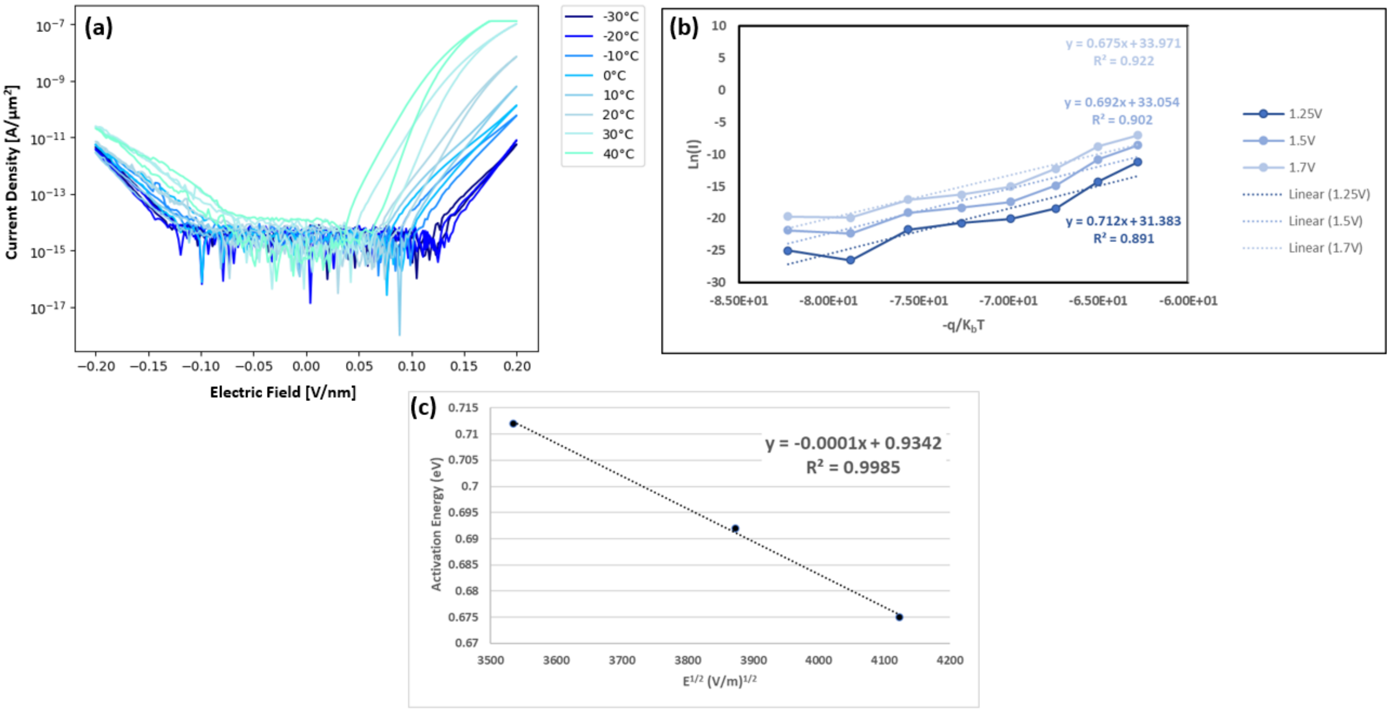

Figure S8. Current-Voltage behavior (a), PF activation energy (b) and trap depth (c) for Ta_2O_5 devices

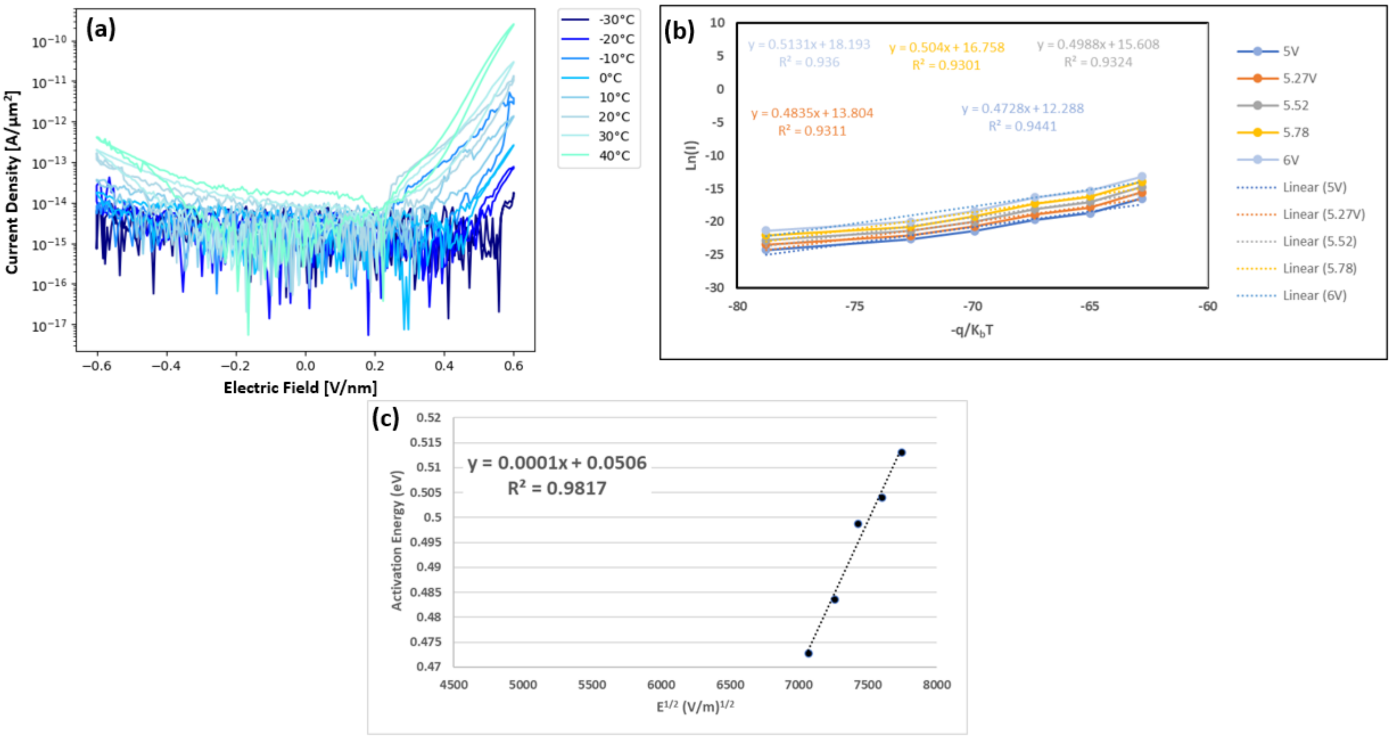

Figure S9. Current-Voltage behavior (a), PF activation energy (b) and trap depth (c) for ZrO_2 devices

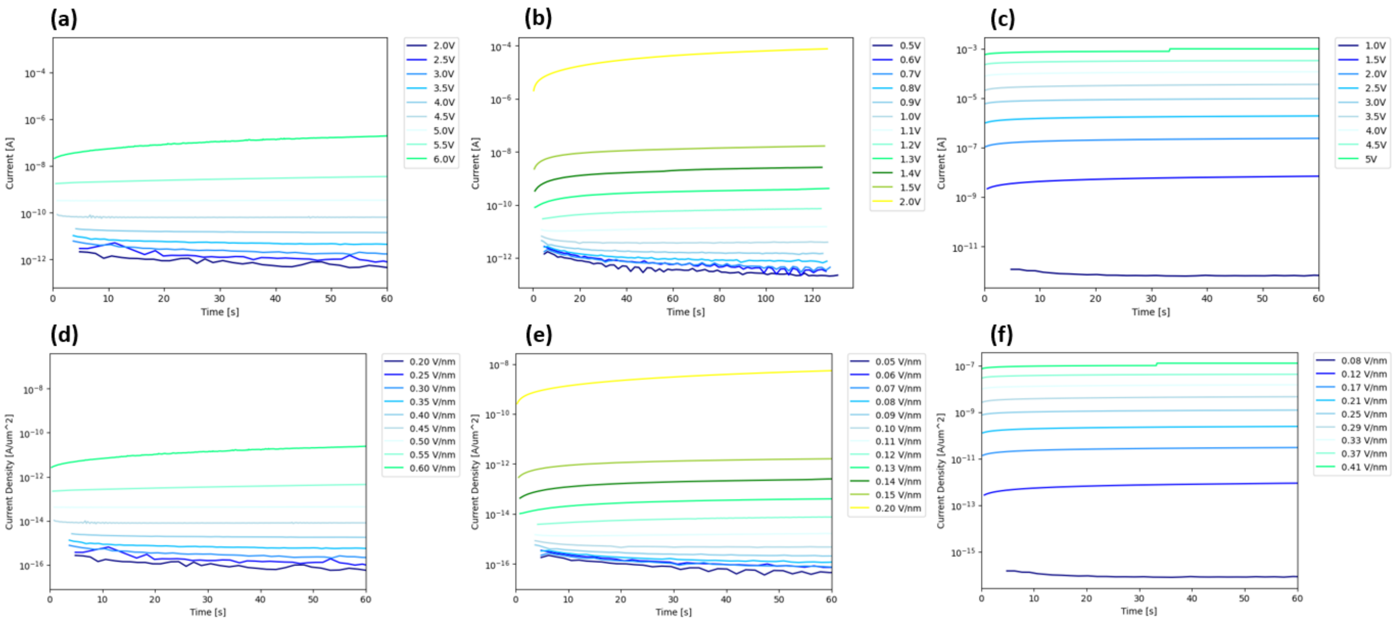

Figure S10. Leakage current for ZrO_2 devices (a), Ta_2O_5 devices (b) and $Zr_3Ta_7O_y$ devices (c) at various voltages over time. Figures (d-f) show current density vs time at the equivalent electric fields to Figures (a-c), respectively

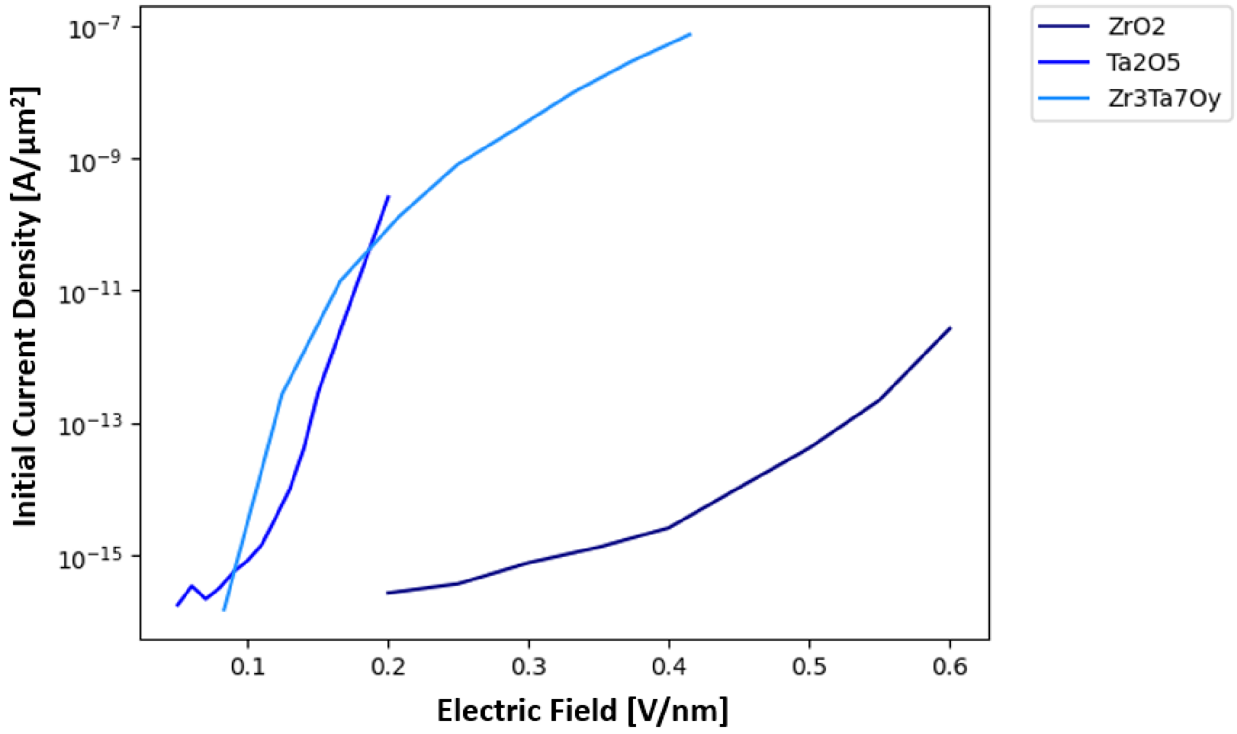

Figure S11. Initial current density of leakage current measurements for Ta_2O_5, ZrO_2 and $Zr_3Ta_7O_y$ devices.

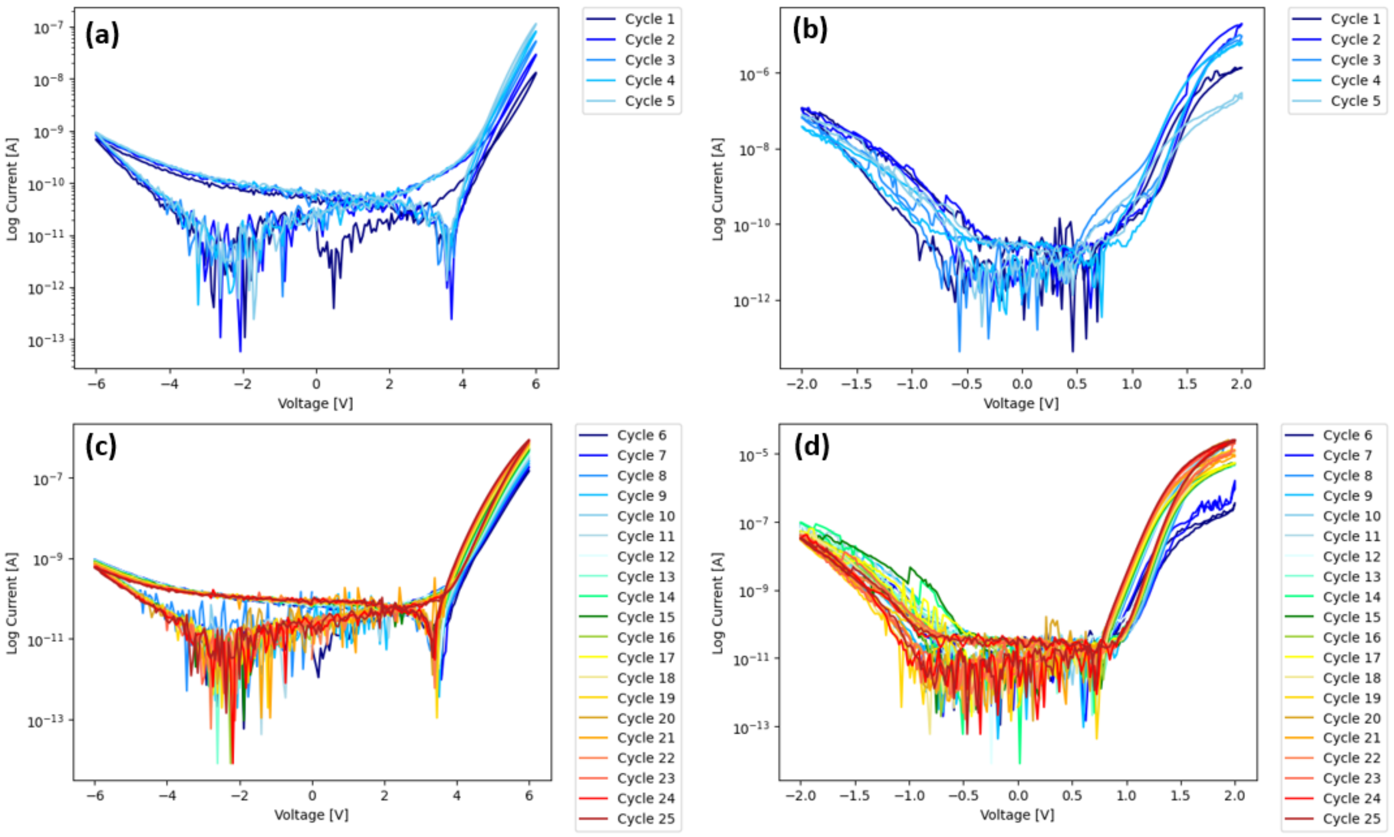

Figure S12. Current-voltage cycling that corresponds to cycled SIMS devices. ZrO_2 devices cycled 25x, (a) and (c), and Ta_2O_5 devices cycled 25x, (b) and (d). Ta_2O_5 cycled device was 9mm away from pristine Ta_2O_5 device while ZrO_2 cycled device was 1mm away from pristine ZrO_2 device. The increased noise in these cycles compared to Figure 3 is attributed to the addition of silver paint to the circuit that, that was used in order to ground the devices.

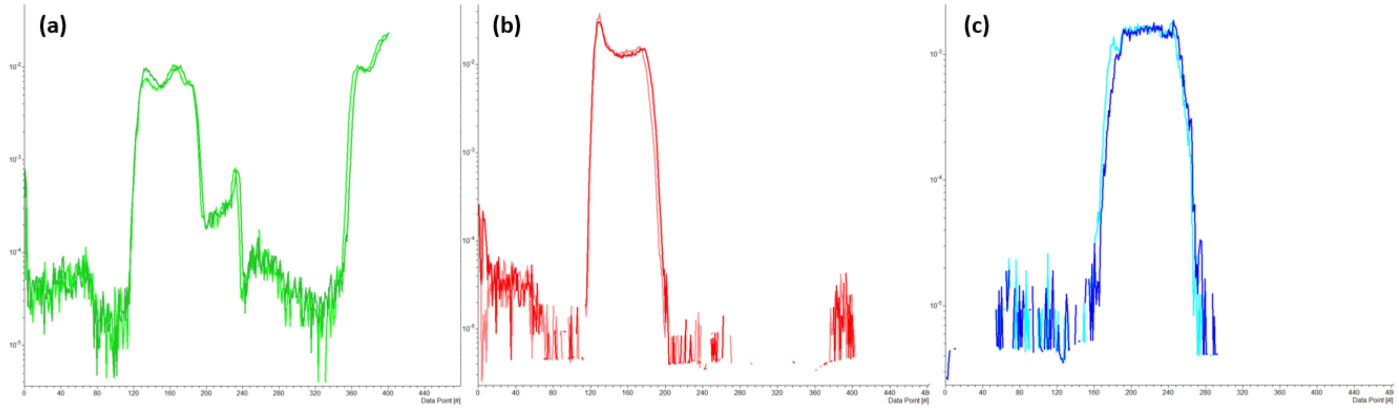

Figure S13. Shows the Cs_2O^+ (a), Zr^+ (b) and Ta^+ (c) normalized to the total signal. Corresponds to Figure 7(a).

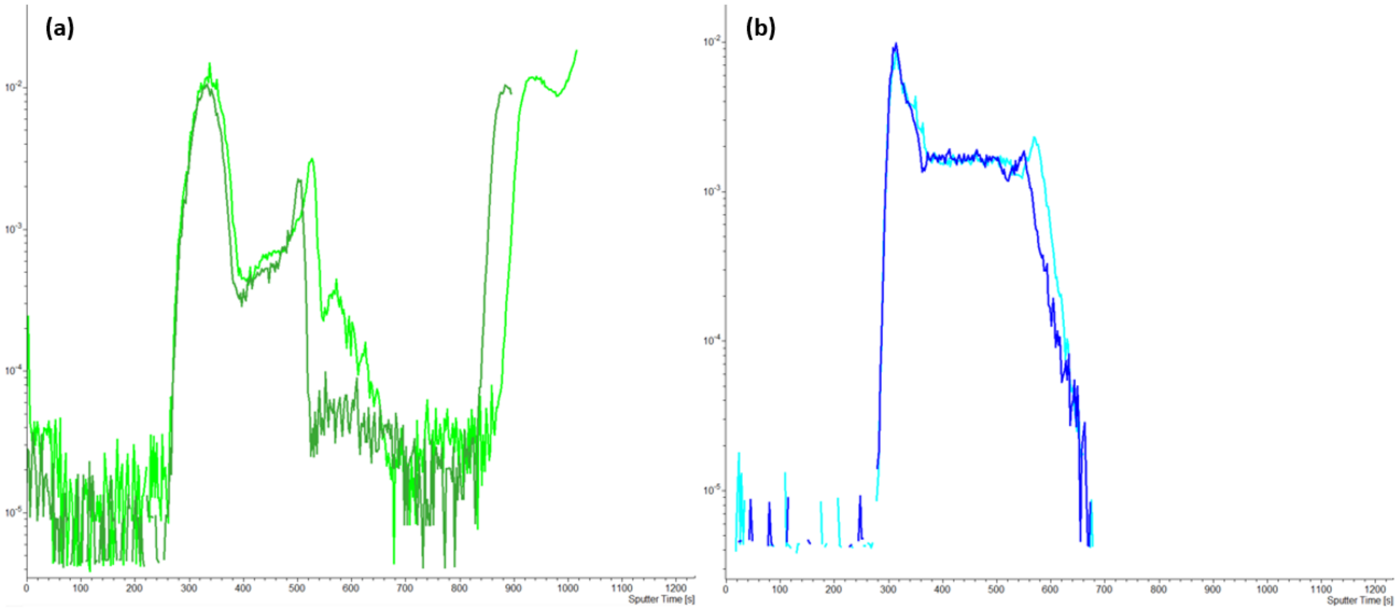

Figure S14. Shows Cs_2O^+ (a) and Ta^+ (b) signals normalized to the total signal. Corresponds to Figure 7(b).
